# Bergamot essential oil improves CUMS‐induced depression‐like behaviour in rats by protecting the plasticity of hippocampal neurons

**DOI:** 10.1111/jcmm.18178

**Published:** 2024-03-30

**Authors:** Jun Chang, Huimin Yang, Xiaoqian Shan, Lan Zhao, Yujiao Li, Zhao Zhang, Joseph Kofi Abankwah, Mingxing Zhang, Yuhong Bian, Yi Guo

**Affiliations:** ^1^ First Teaching Hospital of Tianjin University of Traditional Chinese Medicine Tianjin China; ^2^ National Clinical Research Center for Chinese Medicine Acupuncture and Moxibustion Tianjin China; ^3^ School of Integrative Medicine Tianjin University of Traditional Chinese Medicine Tianjin China; ^4^ Tianjin University of Traditional Chinese Medicine Tianjin China; ^5^ Research Center of Experimental Acupuncture Science Tianjin University of Traditional Chinese Medicine Tianjin China

**Keywords:** aromatherapy, bergamot essential oil, CUMS, depression, hippocampus, inhale, neuron, neuroplasticity, postsynaptic density protein‐95

## Abstract

Bergamot essential oil (BEO) is an extract of the bergamot fruit with significant neuroprotective effect. This study was to investigate the effects and the underlying mechanism of BEO in mitigating depression. GC–MS were used to identify its constituents. Antidepressive properties of BEO were evaluated by sucrose preference test (SPT), force swimming test (FST) and open field test (OFT). Nissl staining was used to determine the number of Nissl bodies in hippocampus (HIPP) of rats. Changes in HIPP dendritic length and dendritic spine density were detected by Golgi‐Cox staining. Immunohistochemistry and Western blot were used to detect the postsynaptic density protein‐95 (PSD‐95) and synaptophysin (SYP) in the HIPP of rats. The enzyme‐linked immunosorbent assay was used to determine the 5‐hydroxytryptamine (5‐HT), insulin‐like growth factor 1 (IGF‐1) and interleukin‐1β (IL‐1β) in the HIPP, serum and cerebrospinal fluid (CSF) of rats. Inhaled BEO significantly improved depressive behaviour in chronic unpredictable mild stress (CUMS) rats. BEO increased Nissl bodies, dendritic length and spine density, PSD‐95 and SYP protein in the HIPP. Additionally, BEO upregulated serum 5‐HT, serum and CSF IGF‐1, while downregulating serum IL‐1β. Collectively, inhaled BEO mitigates depression by protecting the plasticity of hippocampal neurons, hence, providing novel insights into treatment of depression.

## 1 INTRODUCTION

Depression is one of the clinically serious mental diseases, mainly manifested as low mood, slow thinking and even suicidal behaviour.^1^ About 6.79% of patients with depression have suicidal behaviour, which pose a great threat to their physical health and life.^1^ Drug therapy is the main method in treating depression, however, these first‐line antidepressants have little therapeutic effect in one out of three patients, coupled with some adverse reactions.^2^ Thus, safe and effective drugs are urgently needed. Aromatherapy is a non‐invasive green therapy for depression treatment. Bergamot essential oil (BEO) is a natural active ingredient extracted from the aromatic medicine bergamot fruit. Some experiments have reported the antidepressant effects of BEO, yet its mechanism of action has not been elucidated.^3^

Pathogenesis of depression is closely associated with impaired plasticity of hippocampal neurons.^4^ Hence, modulating plasticity of hippocampal neurons may be a novel method to improve depression. In this paper, we planned to establish a rat model of depression by subjecting it to chronic unpredictable mild stress (CUMS), so as to investigate the effects of BEO on depressive‐like behaviours and hippocampal neuroplasticity in CUMS‐induced rats. We aimed at providing scientific basis for the antidepressant properties of BEO and evidence to support its use in the clinical treatment of depression.

## 2 MATERIALS AND METHODS

### 2.1 Chemicals and reagents

Tween 80 (T8360) was gained from Beijing Solarbio Science & Technology Co., Ltd (Beijing, China). Synaptophysin (SYP) Monoclonal Antibody (ab32127) and postsynaptic density protein‐95 (PSD‐95) Polyclonal Antibody (ab18258) and β‐Actin Monoclonal Antibody (ab115777) were provided by abcam (Cambridge, UK). Immunohistochemical staining (PK10006) was provided by Proteintech Group, Inc (Wuhan, China). Golgi‐cox (HTKNS1125‐1) staining solution kit was purchased from HitoBiotec, Inc (Shanghai, China). Toluidine Blue O (T3260) stain was obtained from Sigma‐Aldrich, (Shanghai, USA). The 5‐HT (E‐EL‐0033c) enzyme‐linked immunosorbent assay (ELISA) Kit was purchased from Elabscience Biotechnology Co., Ltd (Wuhan, China). Insulin‐like growth factor 1 (IGF‐1) (EK0377), brain‐derived neurotrophic factor (BDNF) (EK0308), interleukin‐1β (IL‐1β) (EK0393), tumour necrosis factor‐α (TNF‐α) (EK0526) ELISA Kit were obtained from the BOSTER Biological Technology Co., Ltd (Wuhan, China).

### 2.2 Extraction and identification of BEO

Bergamot provided by Zhejiang Jinhua Ru Lai Bergamot Co., Ltd. BEO was extracted using steam distillation and separated by centrifugation. The main compounds of BEO were identified using the gas chromatography–mass spectrometry (GC–MS).^2^ Compound analysis of BEO was performed using a TG‐5MS quartz capillary column (30 m*0.25 mm*0.25 μm). Chromatographic conditions were as follows: the initial temperature was 50°C, retained for 3 min, before increased to 280°C at the rate of 5°C/min and maintained for 5 min (the total analysis time was 54 min and the carrier gas was He). The initial temperature of injection analysis was 50°C, the injection port temperature was 280°C, the injection mode was split mode, the flow rate was 1.0 mL/min, and the split ratio was 100:1. The compounds were qualitatively analysed by GC–MS combined with the retention index of Kovats. The mass spectrum was obtained from the National Institute of Standards (NIST) Mass Spectrometry Library (2014) and Wiley (2008). Total ion spectra of reference substances (C8‐C30) and BEO were determined using GC–MS, and the RI for each compound was calculated and compared with the standard RI to determine the compound.

### 2.3 Animal and experimental design

**FIGURE 1:**
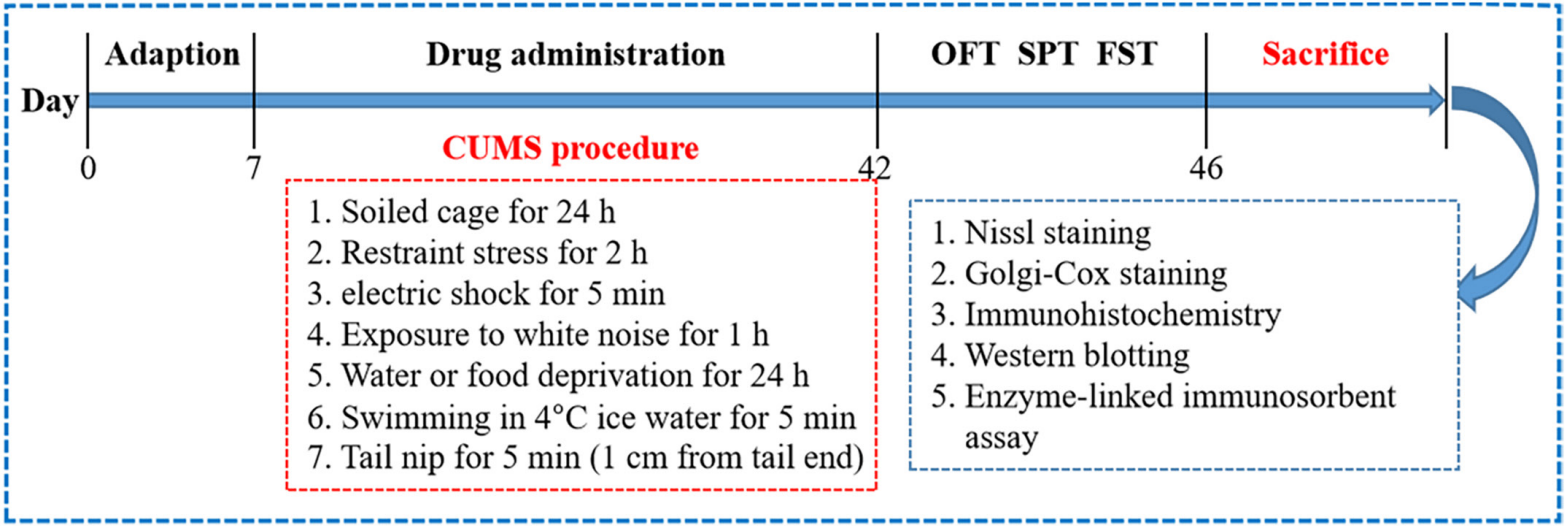
The entire experiment processes including OFT, open field test, SPT, sucrose preference test, FST, forced swimming test from Day 0 to day postsacrifice.

Sprague Dawley rats (170–190 g) were gained from Beijing Vital River Laboratory Animal Technology Co., Ltd. The animals were reared under normal experimental conditions at a room temperature of 24.5 ± 0.5°C, humidity 45%–55%, light/dark cycle of 12 h/12 h, with free drinking water and normal food during the experiment, and adaptive feeding for 1 week before the experiment. The experiments were approved by the Welfare of Experimental Animals of Tianjin University of Chinese Medicine (TCM‐LAEC2015033). The experimental design is shown in Figure 1. The rats were randomly divided into four groups after 1 week of adaptation: control group, CUMS group (1% Tween 80), BEO group (2.5% BEO) and fluoxetine (FLX) group (10 mg/kg). The drugs given to the rats in each group were diluted with normal saline. In this study, we first added 1% Tween 80 to normal saline, which is considered to be CUMS group; then 2.5% BEO is added on top of this, which is considered the BEO group. CUMS rats were administered with 1% Tween 80 via the nasal route using a nebulizer for 1 h. For BEO and FLX groups, rats subjected to CUMS were administered with BEO via the nasal route using a nebulizer for 1 h and FLX was intraperitoneally injected once a day for 5 consecutive weeks, respectively. The CUMS procedure was conducted as previously described with minor modifications.^5^ Rats were subjected to various random stresses for 5 weeks: deprivation of water or food for 24 h, swimming in cold water for 5 min, binding stress for 2 h, nip of tail for 5 min (1 cm from the tail end), white noise for 1 h, electric shock for 5 min and soiled cage for 24 h. All the rats received different types of stress at inconsistent periods each day and did not experience identical stress for 2 days. Therefore, rats were unable to predict the occurrence of stress.

### 2.4 Sucrose preference test

Depressive symptoms were associated with a decreasing percentage of sucrose preference test (SPT) in rats.^6^, ^7^, ^8^ All rats were housed in solitary cages and the test was divided into three phases: ① sucrose water adaptation stage: the rats were given two bottles of 1% sucrose solution (w/v) with the same size and appearance for 24 h; ② water and food deprivation stage: no water and food for 24 h; ③ sucrose water test stage: food and two bottles (with each containing either 1% sucrose water or pure water, respectively) were provided. The rats were made to have free access to food and water at this stage. They were weighed after 12 h, and the measured SPT was obtained using the formulae: (sucrose consumption)/(sucrose consumption + water consumption) × 100%.

### 2.5 Forced swimming test

Depressive behaviour was tested using the forced swimming test (FST) as previously stated.^9^ In this experiment, rats were placed in a columnar bucket (height: 60 cm, diameter: 20 cm) containing water filled up to the 40 cm height of the columnar bucket (water temperature: 25 ± 1°C). This was to ensure that, the rats could only rely on their limbs and tail to swim with their heads out of the water without any support from the base of the bucket. The FST experiment was divided into two parts: (1) the preswimming for 6 min 1 day in advance prior to the main experiment, the rats were taken out, dried with a towel and kept in their respective cages for the day 2 experiment; (2) On the second day, operating in a dimly lit house, the rats were made to swim for 6 min. In order for the rat to float on the water surface, the rat's unilateral or bilateral lower limbs swayed slightly. The main outcome measures were their immobility time and struggling time. The immobility time indicated the time in which the rat did not exude active movement, and only touched the container with the hind limbs or shook their body when necessary to avoid suffocation whiles the struggling time indicated the time the rat climbs a wall or dives.

### 2.6 Open field test

As described in Zhao, we used an open field test (OFT) with minor modifications to assess the effects of BEO on model animals of depression.^10^ OFT was performed in a relatively quiet room, the rat was placed in a fixed position on the bottom of the black open field box, and another person turned on the camera and started timing. The rats' activity was recorded for 6 min, the first 2 min are used to adapt the equipment, and the activity in the last 4 min is analysed.^10^ After each test procedure, the experimental box was cleaned with 75% alcohol, so as to eradicate any leftover visual or chemical cues provided by the latter rat (such as the size, urine and smell of the animal) hence, protecting and ensuring that subsequent test yet to be done are not affected. Observation indicators included total distance, peripheral distance, resting time and activity time.

### 2.7 Nissl staining

Nissl staining was conducted as previously described with minor adjustments.^11^ Per rats were perfused intracardiacally with 0.9% saline followed by 4% paraformaldehyde for 30 min. After fixing the brain, it was embedded in paraffin and cut into 4 μm thick sections through coronal sections. Sections were mounted on glass slides and baked in a 65°C oven for 1 h. The sections were stained with 0.5% Toluidine Blue O for 10 min. All sections were imaged by using a microscope (Nikon, Y‐TV55). The number of Nissl bodies in the HIPP was quantitatively analysed using ImageJ 1.45 software.

### 2.8 Golgi‐cox staining

The dendrite length and spine density in HIPP were measured using the Golgi‐Cox staining solution kit. The brain tissue was stored in the fixative for 48 h, after which it was cut into 2–3 mm pieces. Then it was soaked in staining solution for about half a month away from light, soaked in 80% glacial acetic acid overnight, and put in 30% sucrose. The sheets of brain tissue were then cut into 100 microns and affixed to a slide. After washing and hardening, the tablets are sealed with glycerin gelatin. Finally, panoramic images are obtained by panoramic multilayer scanning. The experiments were carried out in the dark environment.

### 2.9 Western blotting

The HIPP tissue was homogenized in the precooled RIPA lysate. After protein extraction, BCA kit was used to measure protein concentration. It was then separated and moved to the PVDF membrane, incubated overnight at 4°C with primary antibodies against β‐actin (1:10,000, abcam, ab115777) and SYP Antibody (1:10,000, abcam, ab32127) and PSD‐95 Antibody (1:2000, abcam, ab18258). It was washed three times with TBST and incubated with the secondary antibody (1:10,000, Proteintech, SA00001‐7H).

### 2.10 Immunohistochemistry

The brain tissue of 5 μm thickness was cut from HIPP by paraffin section. The paraffin sections were soaked in boiling water solution (0.01 M sodium citrate buffer) for 10 min to extract antigens. The sections were incubated with 5% bovine serum albumin for 1 h at room temperature and incubated with synaptophysin Antibody (1:200, abcam, ab32127) and postsynaptic density protein‐95 Antibody (1:500, abcam, ab18258) overnight at 4°C. On the following day, the sections were incubated with biotinylated anti‐rabbit IgG for 15 min at 37°C and incubated with Horseradish Peroxidase (Proteintech, PK10006) for 30 min at 37°C. Dropped in 15 μl prefabricated DAB working solution and incubated at 37°C temperature for 7.5 min. The sections were stained with haematoxylin for 2 min. The sections were imaged using a microscope (Nikon, Y‐TV55). The image analysis of SYP and PSD‐95‐positive area in the HIPP was quantitatively measured using the ImageJ 1.45 software.

### 2.11 Enzyme‐linked immunosorbent assay

In accordance with manufacturer's indication, the expression of IL‐1β, TNF‐α, 5‐HT and IGF‐1 in the HIPP and cerebrospinal fluid (CSF) and serum of CUMS‐induced rats were tested by using acknowledged kits. The optical density (OD) set at 450 nm was measured using the microplate reader (Thermo Field, USA).

### 2.12 Statistical analysis

Data were presented as mean ± SEM. The SPSS 21.0 software for Windows was used for the Statistical analyses. One‐way ANOVA method was used in multiple comparison between three groups while Student's *t*‐test was used to compare two groups. The *p <* 0.05 was considered statistically significant after comparing the difference.

## 3 RESULTS

### 3.1 GC–MS analysis of BEO

**FIGURE 2:**
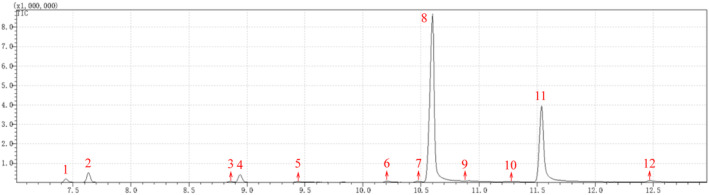
GC–MS ion spectrum of BEO.

GC–MS analysis of BEO revealed the presence of one major constituent (61.69%) at a retention time (Rt) of 10.599 min (Match/R Match 98/98) followed by a peak of lesser abundance (28.5%) at Rt of 11.539 min (Match/R Match 98/98) along with several other minor constituents (Figure 2). In total, 12 compounds were identified, mainly including 1.28% α‐Phellandrene, 3.52% Bicyclo[3.1.0]hexane,4‐methylene‐1‐(1‐methylethyl), 2.8% beta‐Pinene, 61.69% Cyclohexene, 1‐methy1‐4‐(1‐methyl), 28.5% 2,5‐dimethyl‐3‐vinyl‐1,4‐hexadiene (Table 1).

**TABLE 1:** Compounds of the BEO.

Peak	RT (min)	Compounds	Molecular formula	Molecular weight	Area sum%
1	7.437	α‐Phellandrene	C10H16	136.234	1.28
2	7.632	Bicyclo[3.1.0]hexane,4‐methylene‐1‐(1‐methylethyl)	C10H16	136.234	3.52
3	8.856	Bicyclo[3.1.0]hexane, 4‐methyl‐3‐methylene‐1‐(1‐methylethyl)‐ (9CI)	C10H16	136.234	0.32
4	8.941	beta‐Pinene	C10H16	136.234	2.8
5	9.445	beta‐Myrcene	C10H16	136.234	0.3
6	10.205	Cyclohexene,1‐methy1‐4‐(1‐methylethylidene)	C10H16	136.234	0.44
7	10.467	Benzene,1‐methy1‐4‐(1‐methylethy1)‐	C10H16	136.234	0.28
8	10.599	Cyclohexene,1‐methy1‐4‐(1‐methyl)	C10H16	136.234	61.69
9	10.922	Cyclohexene,1‐methy1‐4‐(i‐nethy)	C10H16	136.234	0.25
10	11.254	2,5‐dimethyl‐3‐vinyl‐1,4‐hexadiene	C10H16	136.234	0.15
11	11.539	gamma‐Terpinene	C10H16	136.234	28.5
12	12.475	ALPHA.‐TERPINOLENE	C10H16	136.234	0.47

### 3.2 BEO attenuates CUMS‐induced depressive‐like behaviours in rats

**FIGURE 3:**
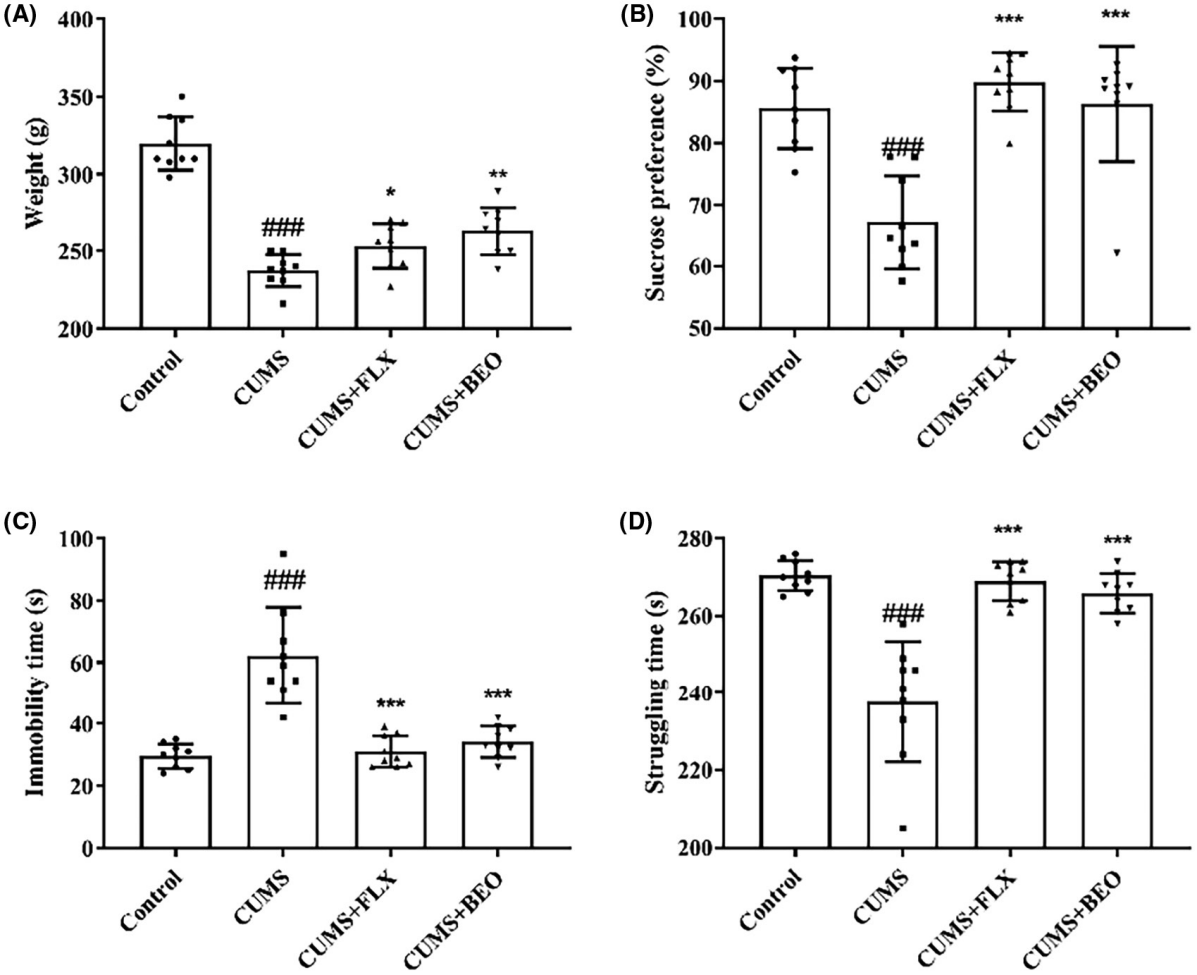
Effects of BEO and FLX on depressive‐like behaviour in the weight (A), SPT (B), and FST (C, D). The values are expressed as means ± SEM for each group (*n* = 9). ^###^compared to the control group, *p* < 0.001. *, **, ***compared to the CUMS group, *p* < 0.05, *p* < 0.01, *p* < 0.001.

After modelling and treatment for 5 weeks, as shown in Figure 3A, compared with the control group, the body weight of the CUMS group was significantly reduced (*p <* 0.001). Compared with the CUMS group, body weight of the rats in the BEO group significantly increased (*p <* 0.01), and the body weight of the rats in the FLX group increased (*p <* 0.05).

The SPT was used to evaluate depressive‐like behaviour in rats. After a five‐week period of CUMS procedure, the consumption of sucrose solution in CUMS group was significantly lower than control group (*p* < 0.001). Five‐week treatment of BEO and FLX significantly increased the consumption of sucrose solution in CUMS rats (BEO, *p* < 0.001; FLX, *p* < 0.001) (Figure 3B). These results indicated that, BEO alleviates depressive‐like emotion in the SPT.

The FST predicted the effectiveness of antidepressants by measuring immobility and struggling time in rats. Compared with the control group, the immobility time of the rats in the CUMS group was significantly prolonged in the FST (*p* < 0.001). Compared with the CUMS group, treatment groups BEO and FLX significantly had an improved immobility time in the FST (BEO, *p* < 0.001; FLX, *p* < 0.001) (Figure 3C). In addition, compared with the control group, the struggling time of the rats in the CUMS group was significantly decreased in the FST (*p* < 0.001). In comparison to the CUMS group, treatment groups BEO and FLX significantly had an increase struggling time in the FST (BEO, *p* < 0.001; FLX, *p* < 0.001) (Figure 3D).

**FIGURE 4:**
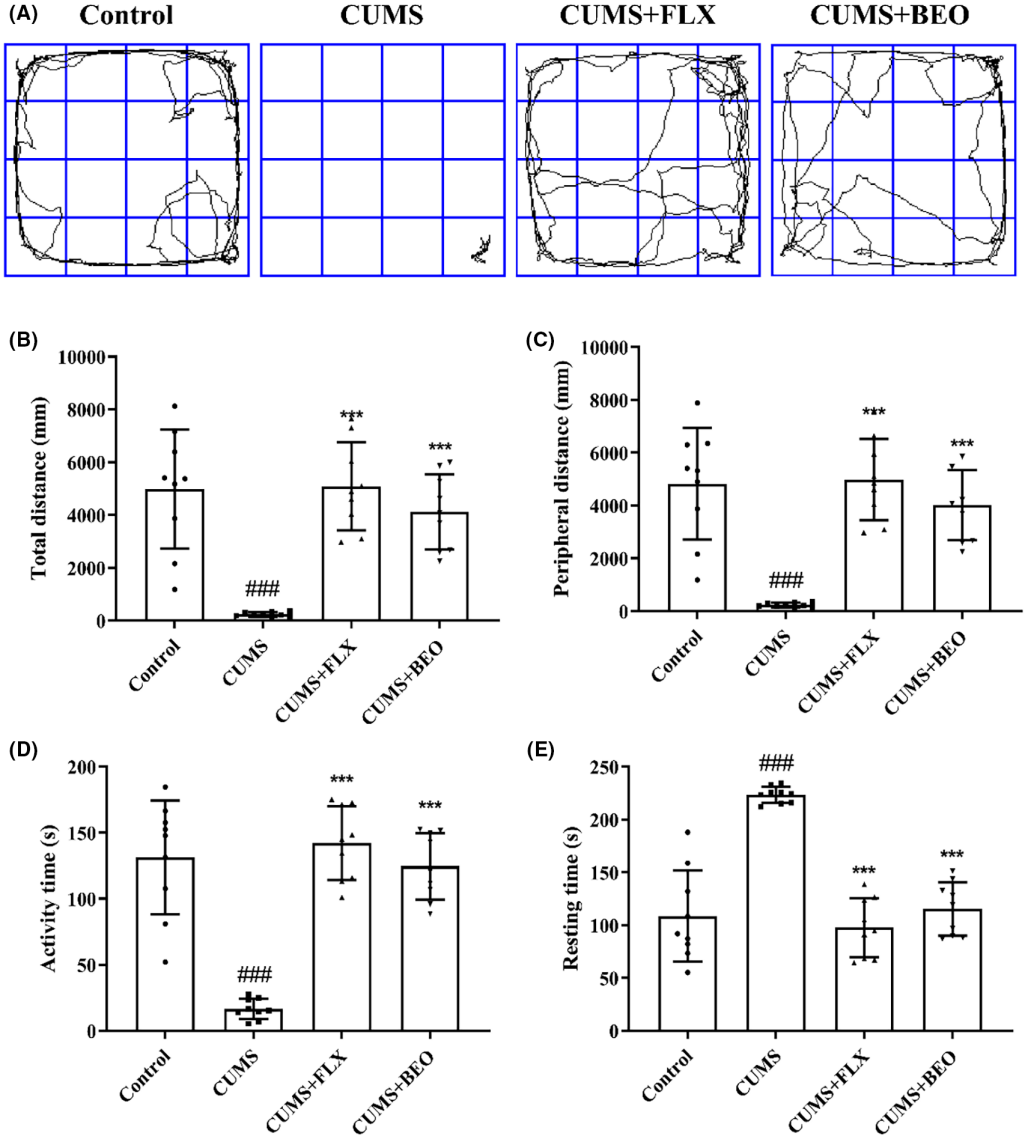
Effects of BEO and FLX on the OFT in CUMS‐induced rats. (A) Trace plot of rats, (B) total distance of movement, (C) peripheral movement distance, (D) activity time, (E) resting time. The values are expressed as means ± SEM for each group (*n* = 9). ^###^compared to the control group, *p* < 0.001. ***compared to the CUMS group, *p* < 0.001.

The effects of BEO on depression rats were further demonstrated by OFT. As shown in Figure 4, CUMS rats showed a significant difference in the trace plot of rats (Figure 4A). Compared with the control group, the total movement distance (Figure 4B), peripheral movement distance (Figure 4C) and activity time (Figure 4D) of the CUMS group were significantly reduced and the resting time (Figure 4E) was significantly increased (*p* < 0.001), suggesting that, the ability of spontaneous activity and exploratory behaviour was weakened. Compared to the CUMS group, BEO and FLX groups significantly reversed the above indicators (BEO, *p* < 0.001; FLX, *p* < 0.001).

### 3.3 Inhaled BEO promotes CUMS‐induced plasticity of hippocampal neurons in rats

#### 3.3.1 BEO increases the number of Nissl bodies in the HIPP

**FIGURE 5:**
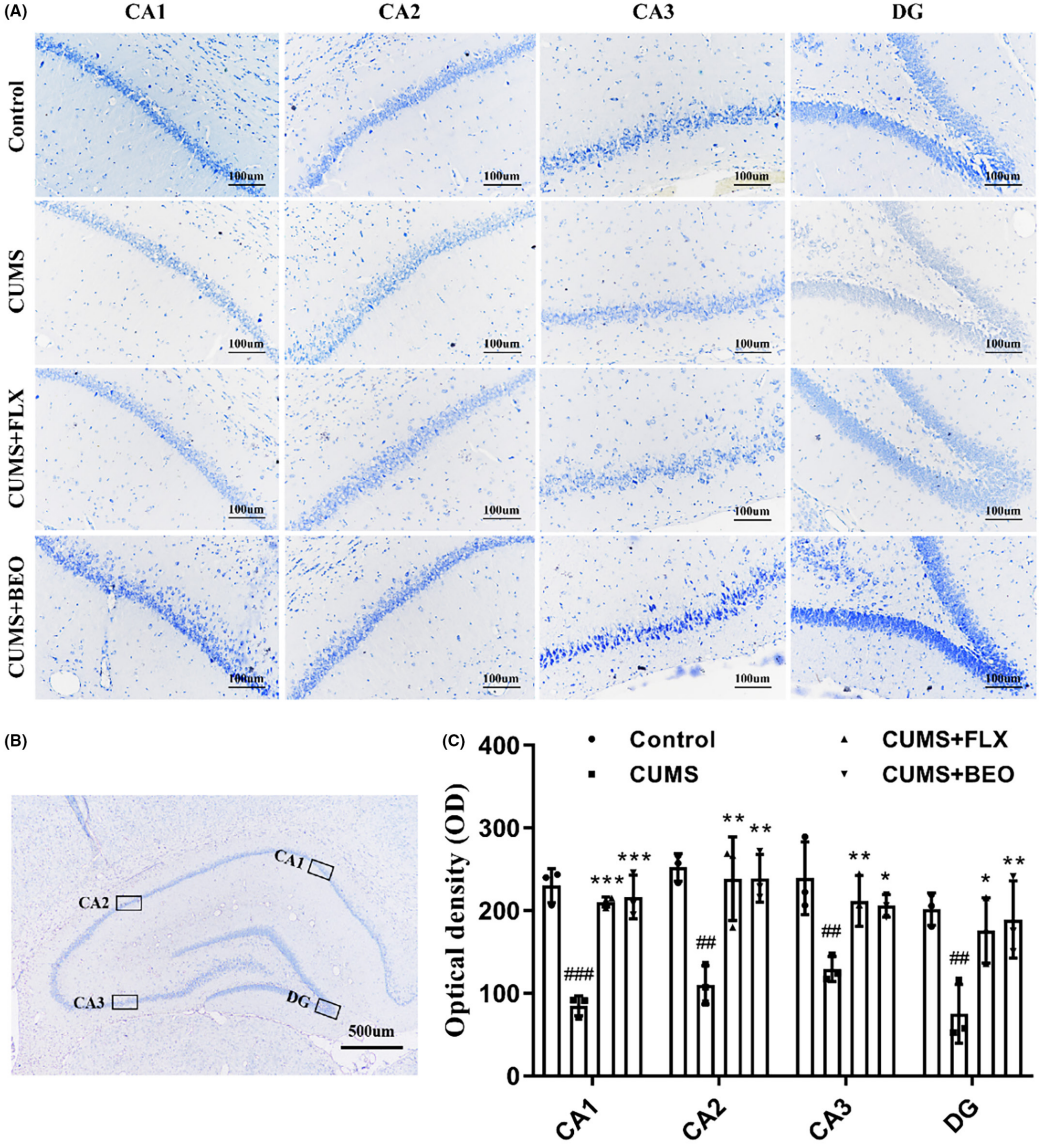
Effects of BEO and FLX on the HIPP Nissl body in CUMS‐induced rats. (A) Representative images of Nissl staining in different regions of the HIPP, (B) schematic illustration of different regions of the HIPP, (C) quantification of optical density values of Nissl bodies in the HIPP. The values are expressed as means ± SEM for each group (*n* = 3). ^##, ###^compared to the control group, *p* < 0.01, *p* < 0.001. *, **, ***compared to the CUMS group, *p* < 0.05, *p* < 0.01, *p* < 0.001.

Depression is closely related to the number of Nissl body. As shown in Figure 5, compared with the control group, the number of Nissl bodies in each region of the HIPP belonging to the CUMS group significantly decreased (CA1, *p* < 0.001; CA2, *p* < 0.01; CA3, *p* < 0.001; DG, *p* < 0.01). Compared with the CUMS group, BEO significantly increased the number of Nissl bodies in each region of the HIPP (CA1, *p* < 0.001; CA2, *p* < 0.01; CA3, *p* < 0.001; DG, *p* < 0.01) of the rats, while rats in the FLX group showed a significant increase in the number of Nissl bodies in each region of the HIPP (CA1, *p* < 0.001; CA2, *p* < 0.01; CA3, *p* < 0.001; DG, *p* < 0.05).

#### 3.3.2 BEO augments dendritic length and spines density in HIPP of rats with CUMS

**FIGURE 6:**
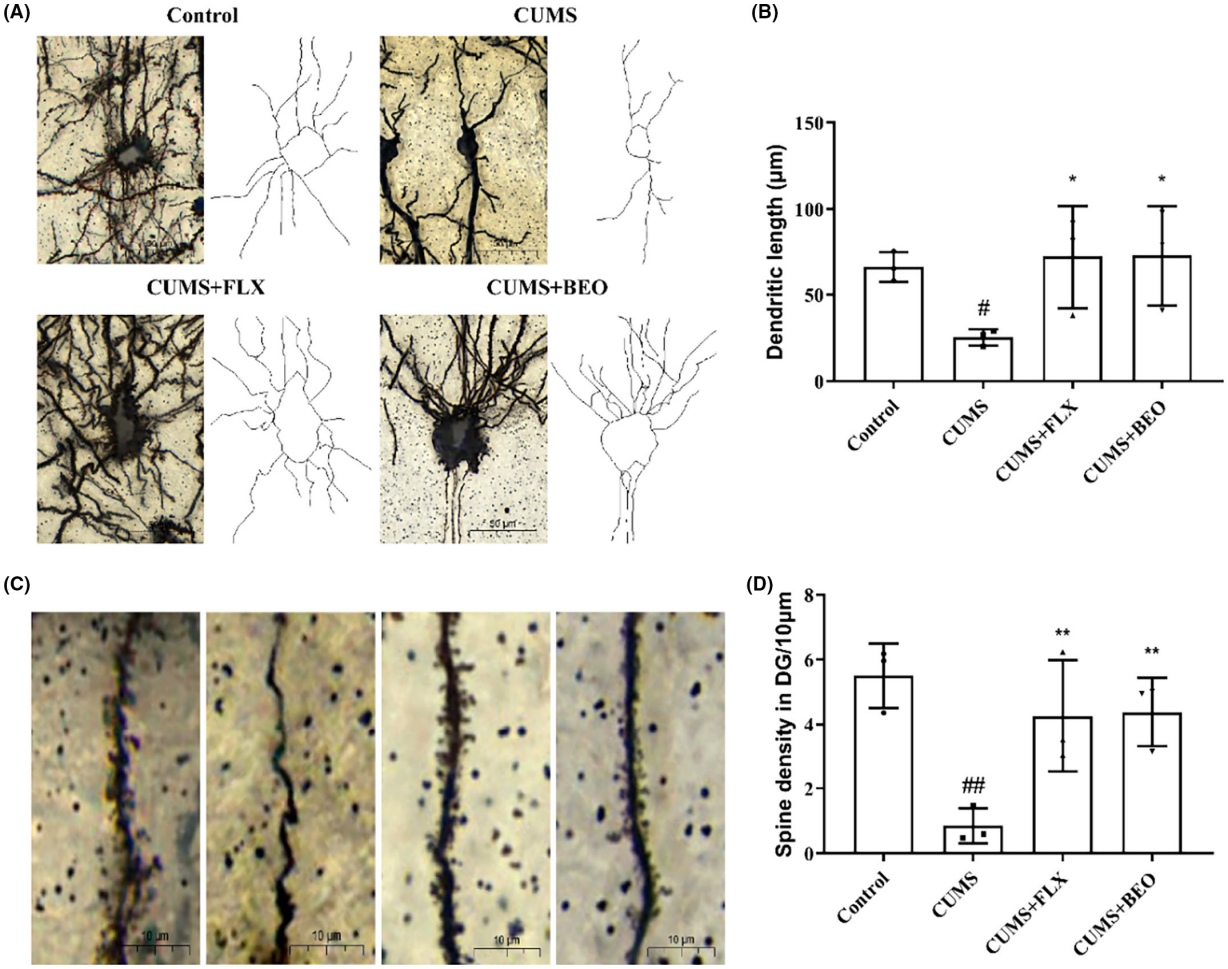
Effect of BEO and FLX on dendritic length and spine density in HIPP. (A) Representative images of dendritic length in DG regions of the HIPP, (B) quantification of dendritic length in the HIPP, (C) representative images of dendritic spine density in DG regions of the HIPP, (D) quantification of dendritic spine density in DG regions of the HIPP. The values are expressed as means ± SEM for each group (*n* = 1, repeat three times each). ^#, ##^compared to the control group, *p* < 0.05, *p* < 0.01. *, **compared to the CUMS group, *p* < 0.05, *p* < 0.01.

The dendritic length and spine density in the HIPP of each group were detected using the Golgi staining method. We demonstrated that, BEO affected HIPP dendritic length and spine density in depression rats. The results of dendritic length shown in Figure 6A,B, indicated that, the length of hippocampal dendrites in CUMS group was significantly shorter than the control group (*p* < 0.05). After BEO and FLX treatment, the length of HIPP dendrites in CUMS group increased significantly (BEO, *p* < 0.05; FLX, *p* < 0.05). Golgi‐Cox staining images showed that, the dendritic spine density in HIPP in CUMS‐induced rats were significantly reduced than the control group (*p* < 0.01), whereas BEO and FLX reversed the reduction of dendritic spine density induced by CUMS (BEO, *p* < 0.01; FLX, *p* < 0.01) (Figure 6C,D).

#### 3.3.3 BEO reverses PSD‐95 and SYP decline in HIPP of rats with CUMS

**FIGURE 7:**
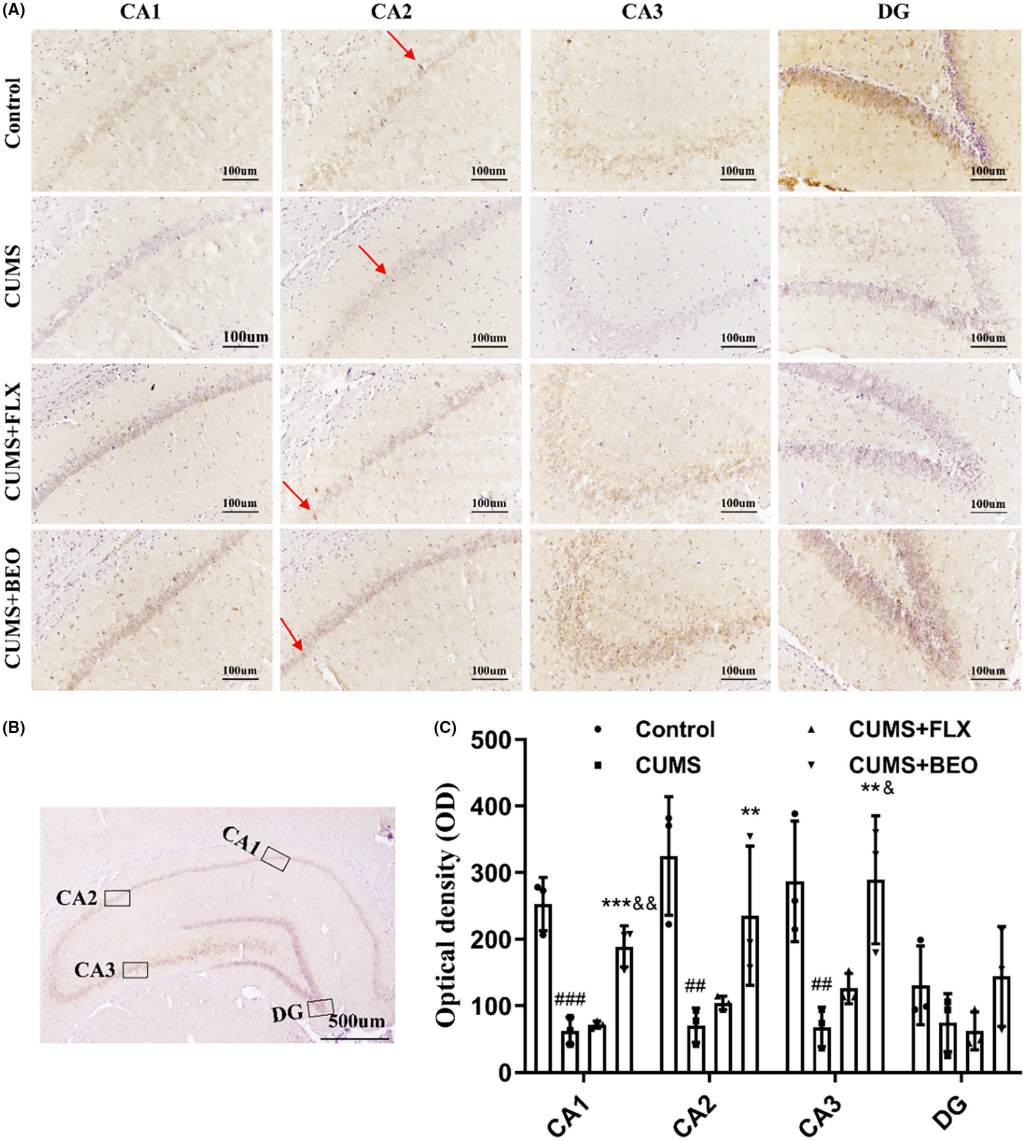
Effects of BEO and FLX on the HIPP PSD‐95 in CUMS‐induced rats. (A) Representative images of PSD‐95 in different regions of the HIPP, (B) schematic illustration of different regions of the HIPP, (C) quantification of optical density values of PSD‐95 in the HIPP. The values are expressed as means ± SEM for each group (*n* = 3). ^##, ###^compared to the control group, *p* < 0.01, *p* < 0.001. **, ***compared to the CUMS group, *p* < 0.01, *p* < 0.001. ^&&^compared to the FLX group, *p* < 0.01.

The expression of key proteins in the hippocampal neuroplasticity of rats in each group was detected by IHC and WB. As shown in Figure 7, compared with the control group, the expression of PSD‐95 in the CA1, CA2 and CA3 regions of the CUMS group was significantly decreased (CA1, *p* < 0.001; CA2, *p* < 0.01; CA3, *p* < 0.001). BEO significantly increased the expression of PSD‐95 in CA1, CA2 and CA3 regions in CUMS rats (CA1, *p* < 0.001; CA2, *p* < 0.01; CA3, *p* < 0.01). Compared with the FLX group, the expression of PSD‐95 in the CA1 region of the BEO group was increased (*p* < 0.01) (Figure 7C).

**FIGURE 8:**
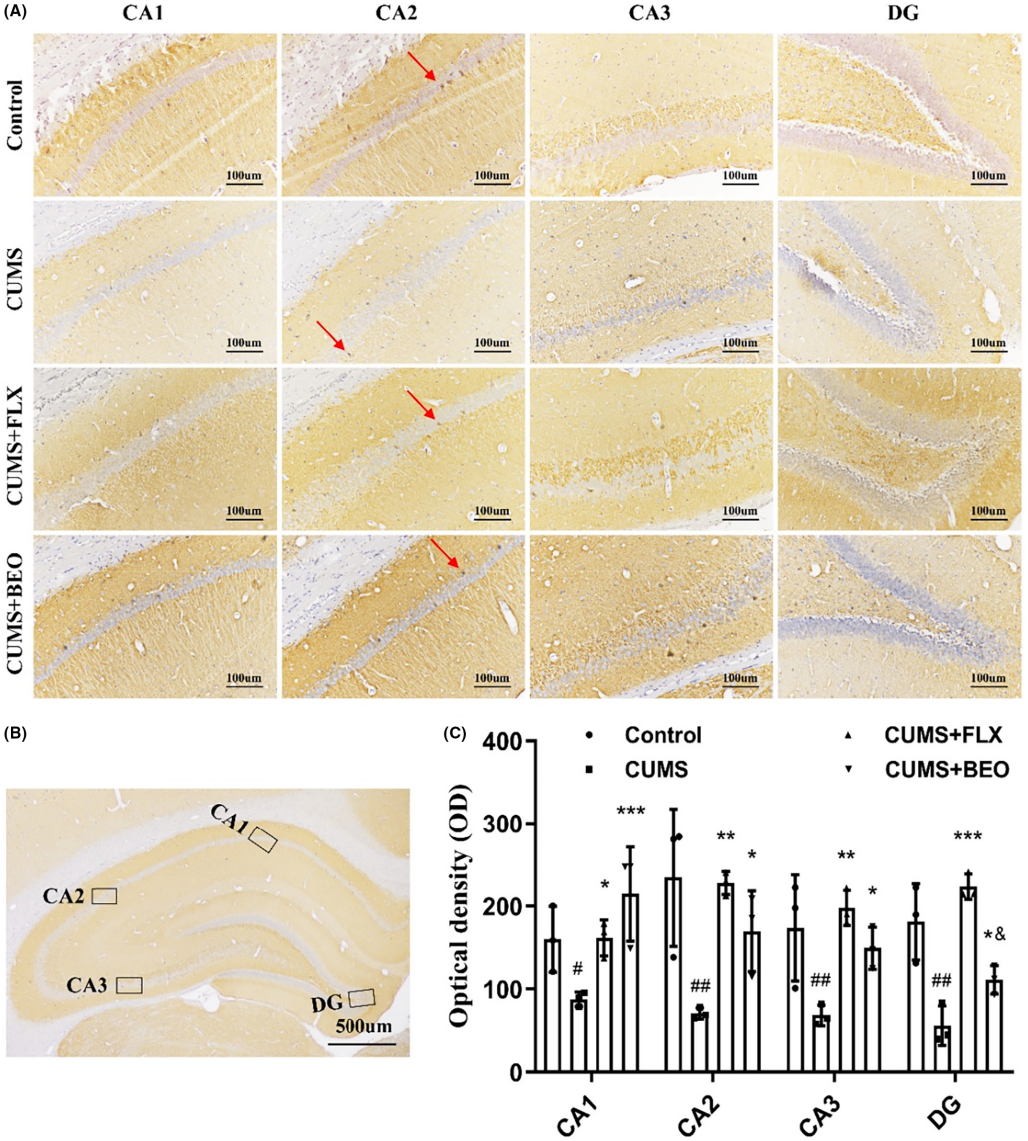
Effects of BEO and FLX on the HIPP SYP in CUMS‐induced rats. (A) Representative images of SYP in different regions of the HIPP, (B) schematic illustration of different regions of the HIPP, (C) quantification of optical density values of SYP in the HIPP. The values are expressed as means ± SEM for each group (*n* = 3). ^#, ##^compared to the control group, *p* < 0.05, *p* < 0.01. *, **, ***compared to the CUMS group, *p* < 0.05, *p* < 0.01, *p* < 0.001. ^&^compared to the FLX group, *p* < 0.05.

As shown in Figure 8, compared with the control group, the expression of SYP in the HIPP each region of the CUMS group was significantly decreased (CA1, *p* < 0.01; CA2, *p* < 0.01; CA3, *p* < 0.001; DG, *p* < 0.01). Compared with the CUMS group, the BEO group increased the expression of SYP in the HIPP each region of rats (CA1, *p* < 0.001; CA2, *p* < 0.05; CA3, *p* < 0.01; DG, *p* < 0.05), rats in the FLX group showed a significant increase in the HIPP each region of rats (CA1, *p* < 0.01; CA2, *p* < 0.01; CA3, *p* < 0.001; DG, *p* < 0.001). Compared with the FLX group, the expression of SYP protein in the CA3 and DG regions of the BEO group was decreased (CA3, *p* < 0.05; DG, *p* < 0.05) (Figure 8C).

**FIGURE 9:**
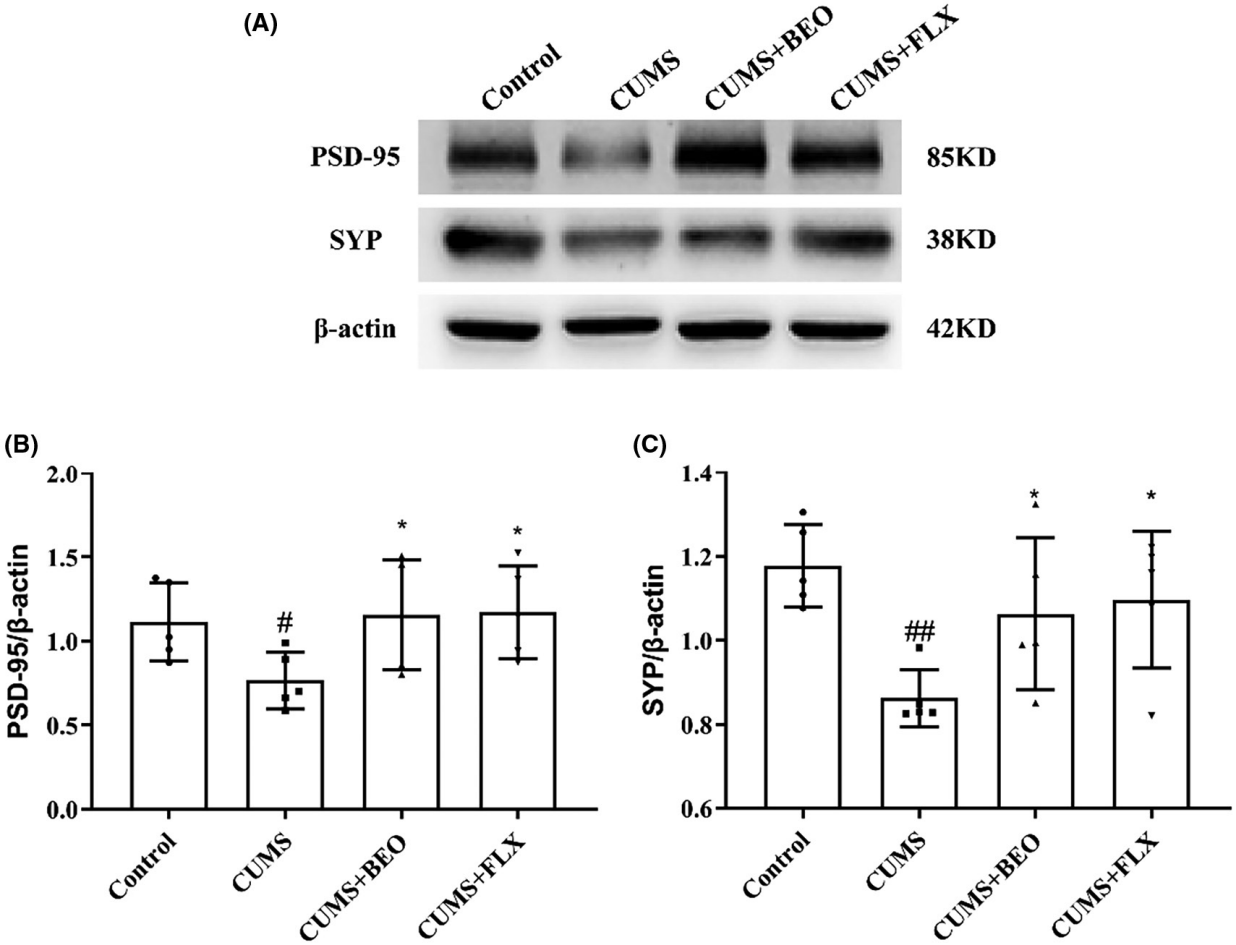
Effects of BEO and FLX on the HIPP PSD‐95 and SYP in CUMS‐induced rats. (A) Western blot analysis of PSD95 and SYP levels in the HIPP, with β‐Actin as the loading control. (B, C) Quantification of PSD95 and SYP levels from the immunoblot experiment in panel A. The values are expressed as means ± SEM for each group (*n* = 5). ^#, ##^compared to the control group, *p* < 0.05, *p* < 0.01. *compared to the CUMS group, *p* < 0.05.

We used WB to detect the differences of PSD‐95 and SYP proteins in the HIPP of CUMS rats. As shown in Figure 9, compared with the control group, the expression of PSD‐95 and SYP in the HIPP of the CUMS group were significantly decreased (PSD‐95, *p* < 0.05; SYP, *p* < 0.01). The expression of HIPP PSD‐95 was significantly increased after BEO and FLX treatment (BEO, *p* < 0.05; FLX, *p* < 0.05) (Figure 9B). Analogously, compared to the CUMS group, the expression of HIPP SYP in the BEO and FLX groups were significantly increased (BEO, *p* < 0.05; FLX, *p* < 0.05) (Figure 9C).

### 3.4 Effects of BEO on 5‐HT, IGF‐1, BDNF, IL‐1β and TNF‐α in CUMS rats

**FIGURE 10:**
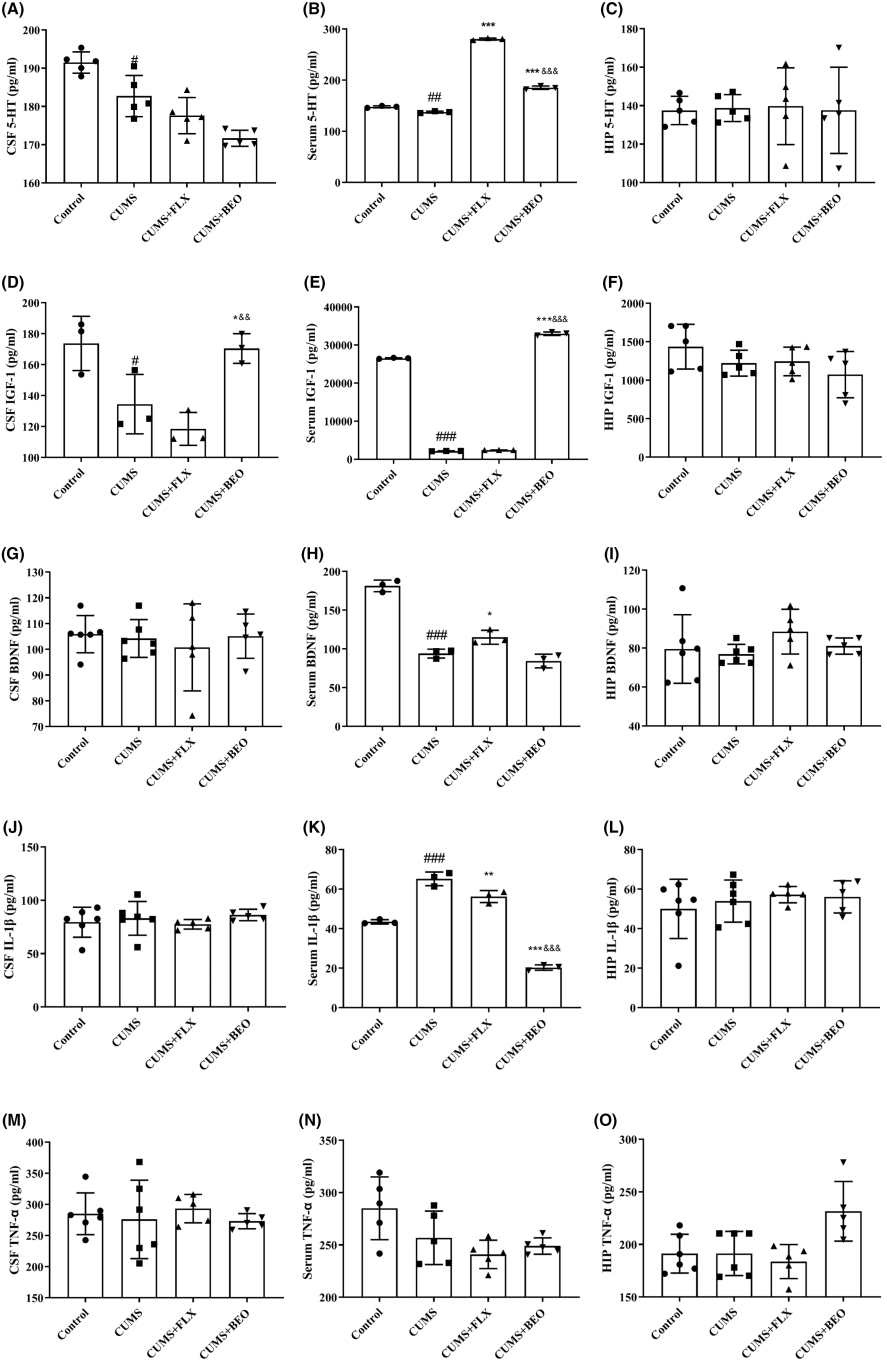
Effects of BEO and FLX on 5‐HT, IGF‐1, BDNF, IL‐1β and TNF‐α in CUMS rats. (A) CSF. (B) Serum. (C) HIP. (D) CSF. (E) Serum. (F) HIP. (G) CSF. (H) Serum. (I) HIP. (J) CSF. (K) Serum. (L) HIP. (M) CSF. (N) Serum. (0) HIP. The values are expressed as means ± SEM for each group (*n* = 3–6). ^#, ##, ###^compared to the control group, *p* < 0.05, *p* < 0.01, *p* < 0.001. *, **, ***compared to the CUMS group, *p* < 0.05, *p* < 0.01, *p* < 0.001. ^&&, &&&^compared to the FLX group, *p* < 0.01, *p* < 0.001.

To confirm effects of BEO on 5‐HT, IGF‐1, BDNF, IL‐1β and TNF‐α, the ELISA kits were used to assess the levels of 5‐HT, IGF‐1, BDNF, IL‐1β and TNF‐α in the CSF serum, and HIPP of rats. The results showed in Figure 10A,B, indicate that, the expression of 5‐HT in the serum and CSF of the CUMS group were significantly decreased, compared with the control group (serum, *p* < 0.05; CSF, *p* < 0.05). After 5 weeks of treatment, the expression of 5‐HT in serum of CUMS rats was significantly increased in BEO and FLX groups (BEO, *p* < 0.001; FLX, *p* < 0.001); the level of 5‐HT in BEO group was significantly lower than FLX group (*p* < 0.001) (Figure 10B). BEO had no significant effect on 5‐HT in the HIPP of CUMS rats (*p* > 0.05) (Figure 10C).

As described in Figure 10D,E, after 5 weeks of random stress, the expression of IGF‐1 in serum and CSF of CUMS group was significantly decreased (serum, *p* < 0.001; CSF, *p* < 0.05). Interestingly, IGF‐1 levels in CUMS serum and CSF were significantly increased after BEO treatment (serum, *p* < 0.001; CSF, *p* < 0.05). Moreover, the expression of IGF‐1 in serum and CSF in BEO group was significantly higher than FLX group (serum, *p* < 0.001; CSF, *p* < 0.01). BEO had no significant effect on IGF‐1 in the HIPP of CUMS rats (*p* > 0.05) (Figure 10F).

In Figure 10H, compared with the control group, BDNF levels in the serum of the CUMS group were significantly decreased (*P* < 0.001). The expression of BDNF in the serum of the FLX group were significantly increased compared to the CUMS group (*p* < 0.05). Nevertheless, BEO had no obvious effect on BDNF in serum, CSF and HIPP of CUMS rats (*p* > 0.05) (Figure 10G–I). It is suggested that, BEO may not be related to BDNF in promoting neuroplasticity and antidepressant mechanism.

In Figure 10K, we found that, CUMS significantly increased serum IL‐1β levels in rats (*p* < 0.001). Serum IL‐1β levels in BEO and FLX groups were significantly lower than those in CUMS (BEO, *p* < 0.001; FLX, *p* < 0.01). Moreover, serum IL‐1β level in BEO group was better than FLX group (*p* < 0.001). However, BEO had no significant effect on IL‐1β in the HIPP and CSF of CUMS rats (*p* > 0.05) (Figure 10J,L). BEO had no significant effect on TNF‐α in serum, CSF and HIPP of CUMS rats (*p* > 0.05) (Figure 10M–O).

## 4 DISCUSSION

Depression is a major disease endangering human health with a high rate of death and disability, but lack effective drug treatment. Bergamot has a long history of treating depression, and modern pharmacological studies have found that, bergamot extraction (BEO) can cross the blood–brain barrier, has good permeability, rapid metabolism, good safety profile and very effective for treating mild–moderate depression.^9^, ^12^ For example, a clinical study found that, BEO inhaled for 30 min daily for 16 days of intervention increased plasma 5‐HT and reduced depression scale and health questionnaire scores in elderly depression.^13^ Similarly, a study found that, an aromatic spray consisting of BEO and lavender essential oil inhaled for 4 weeks was effective in reducing depression, anxiety and stress in older adults.^14^ Animal experiments found that 2.5% BEO inhaled for 14 days reduced the immobility time of FST and increased HIPP BDNF levels in depression‐like rats.^15^ Similar study found that, 2.5% BEO administered intraperitoneally, significantly improved anxiety‐like symptoms and inhibited HPA axis activity,^16^ however, the detailed mechanism is not fully understood. In conclusion, BEO is effective in the treatment of depression with no significant adverse effects.

Behavioural experiments are commonly used to assess whether a drug is an antidepressant, and we chose SPT, FST and OFT and body weight to assess the antidepressant effects of BEO. The results revealed that, CUMS‐induced a decrease in sucrose preference rate, immobility time and distance to exercise and body weight in rats, suggesting a lack of exploration and activity ability, yet BEO inhalation significantly reversed the CUMS‐induced depression‐like behaviour in rats, indicating that, BEO has a significant antidepressant effect, but its mechanism of action is unclear. Therefore, we further explored the mechanisms by which BEO ameliorates depression‐like behaviours.

Plasticity of hippocampal neurons is the brain's ability to make neurobiological changes in response to external stimuli or internal stresses. The HIPP plays a key role in emotion, cognition and memory.^17^, ^18^ Recent studies have found that, impaired plasticity of hippocampal neurons contributes to the pathogenesis of depression.^4^ CUMS lead to impairment of plasticity of hippocampal neurons and depressive‐like behaviours in animal models,^5^, ^18^, ^19^ hence promoting hippocampal neuroplasticity improved CUMS‐induced depressive‐like mood.^18^ PSD‐95 and SYP are important markers of neuroplasticity in the HIPP. Large evidence postulates that expression of PSD‐95 and SYP in the HIPP of rats with depression are decreased; conversely, increasing PSD‐95 and SYP in the HIPP alleviated depressive symptoms.^20^, ^21^ Accordingly, boosting hippocampal neuroplasticity maybe an attractive method to improve depression.

Nissl bodies are considered to be one of the hallmark features of neuronal damage and strongly associated with impaired neuroplasticity.^11^ Our results showed that, inhaled BEO increased the number of Nissl bodies in the HIPP of CUMS‐induced rats. The autopsy results of patients with depression showed that, the dendrite length and density in the HIPP of patients with depression were decreased.^20^ In our study, our study found that CUMS caused a decrease in dendrite length and spinal density in rats. This is similar to the results of some studies in recent years, CUMS induced the decrease of dendrite length and spinal density in the brain region of depressed rats.^6^, ^22^, ^23^, ^24^, ^25^ Hei et al. found that chronic mild stress increased the density of synpase and small spine in inner and outer in DG,^26^ which may be strongly related to different types of animal models, because our research object is rats, while Hei et al.'s research object is mice. Secondly, we believe that the difference of stress mode and time is also an important reason affecting the experimental results.^25^ Moreover, we also found inhaling BEO increased dendritic length and spine density in the HIPP of CUMS‐induced rats.

Further research showed that, CUMS decreases HIPP plasticity‐related proteins (PSD‐95, SYP), suggesting that CUMS reduced hippocampal neuroplasticity. Conversely inhaled BEO elevated HIPP PSD‐95 and SYP proteins, consequently improving depressive‐like symptoms. In this study, IHC and WB results showed that, the expressions of PSD‐95 and SYP protein was significantly decreased in the HIPP of CUMS‐induced rats. Interestingly, PSD‐95 and SYP was significantly upregulated in CUMS‐induced rats after inhaling BEO. All in all, BEO alleviated CUMS‐induced impairment of plasticity of hippocampal neuron**s** in rats. However, the mechanism of action needs to be further elucidated.

Several studies have shown that, abnormal levels of 5‐HT, IGF‐1, TNF‐α and IL‐1β are closely related to impaired neuroplasticity. Magnetic resonance imaging found that, reduction in synaptic density and volume of HIPP in depressed patients are closely associated with the changes of 5‐HT.^27^ On the contrary, Ketamine and some traditional Chinese medicines enhanced neuroplasticity by increasing 5‐HT to treat depression.^27^ We also found that, inhaled BEO significantly augmented 5‐HT in serum, had no significant effect on 5‐HT in CSF and HIPP, which was similar to previous findings.^10^, ^17^, ^28^ The reason why BEO has no significant effect on HIPP 5‐HT may be related to the small number of brain tissue types examined and the dynamic changes of 5‐HT. Some studies have found that, drugs have no effect on HIPP 5‐HT, but affect 5‐HT in other tissues, such as prefrontal cortex (PFC)^10^ and serum.^17^ Moreover, IGF‐1 is an ancient neurotrophic hormone that plays an important role in CNS development and maturation. IGF‐1 transmits signals through its receptor (IGF‐1R) and classical signalling pathways (such as PI3K‐Akt), which affect the CNS plasticity.^27^ Our results indicated that, IGF‐1 decreased significantly in serum and CSF of CUMS‐induced rats, while inhaled BEO significantly increased IGF‐1 in serum and CSF of CUMS‐induced rats. HIPP IGF‐1 was tended to decrease, but the difference was not statistically significant, which is familiar with previous studies.^29^ In addition, inflammatory cytokines are associated with impaired neuroplasticity. TNF‐α and IL‐1β affect BDNF receptor (Trk‐β) phosphorylation, interfere with BDNF signalling and attenuate downstream phospholipase Cγ1 and activation of extracellular regulated signalling kinase, thereby reducing neuroplasticity in HIPP.^30^ We found that, IL‐1β levels in the serum were markedly upregulated in CUMS‐induced rats, while inhaled BEO reduced serum IL‐1β, had no significant effect on IL‐1β in the CSF and HIPP, which is consistent with previous research.^31^ BEO had no significant effect on TNF‐α in the serum, CSF and HIPP.

There are some limitations in our study, it is not clear whether BEO enters the brain tissue through the nasal mucosa or through the blood–brain barrier to exert antidepressant effects, the composition of BEO in blood and CSF can be clarified by GC–MS technique in future. In addition, the mechanism of study was not in‐depth, and in vitro experiments, gene knockout and gene overexpression experiments should be conducted in the future to elucidate the mechanism of BEO on neuroplasticity in the HIPP of rat model for depression.

## 5 CONCLUSION

This study demonstrated that, BEO exerts antidepressant effects via protecting the plasticity of hippocampal neurons, and the underlying mechanism may be related to increasing concentration of 5‐HT in serum, IGF‐1 in serum and CSF, and decreasing concentration of IL‐1β in serum. This data furnishes new insight to its possible therapeutic use against depression.

## AUTHOR CONTRIBUTIONS

**Jun Chang:** Conceptualization (equal); data curation (equal); methodology (equal); project administration (equal); resources (equal); writing – original draft (equal). **Huimin Yang:** Data curation (equal); formal analysis (equal); methodology (equal). **Xiaoqian Shan:** Data curation (equal). **Lan Zhao:** Methodology (equal). **Yujiao Li:** Project administration (equal). **Zhao Zhang:** Project administration (equal). **Joseph Kofi Abankwah:** Methodology (equal). **Mingxing Zhang:** Conceptualization (equal); formal analysis (equal); project administration (equal); writing – review and editing (equal). **Yuhong Bian:** Formal analysis (equal); project administration (equal); supervision (equal); writing – review and editing (equal). **Yi Guo:** Formal analysis (equal); project administration (equal); supervision (equal); writing – review and editing (equal).

## CONFLICT OF INTEREST STATEMENT

All authors declare no conflict of interest.

## Data Availability

Data can be accessed by emailing the corresponding authors.
